# Identification and evolutionary analysis of long non-coding RNAs in zebra finch

**DOI:** 10.1186/s12864-017-3506-z

**Published:** 2017-01-31

**Authors:** Chih-Kuan Chen, Chun-Ping Yu, Sung-Chou Li, Siao-Man Wu, Mei-Yeh Jade Lu, Yi-Hua Chen, Di-Rong Chen, Chen Siang Ng, Chau-Ti Ting, Wen-Hsiung Li

**Affiliations:** 1 Institute of Ecology and Evolutionary Biology, National Taiwan University, Taipei, 10617 Taiwan; 2 Biodiversity Research Center, Academia Sinica, Taipei, 11529 Taiwan; 3 Department of Medical Research, Genomics and Proteomics Core Laboratory, Kaohsiung Chang Gung Memorial Hospital and Chang Gung University College of Medicine, Kaohsiung, 83301 Taiwan; 4 Institute of Molecular and Cellular Biology & Department of Life Science, National Tsing Hua University, Hsinchu, 30013 Taiwan; 5 Department of Life Science & Genome and Systems Biology Degree Program, National Taiwan University, Taipei, 10617 Taiwan; 6 Research Center for Developmental Biology and Regenerative Medicine, National Taiwan University, Taipei, 10617 Taiwan; 7 Center for the Integrative and Evolutionary Galliformes Genomics (iEGG Center), National Chung Hsing University, Taichung, 40227 Taiwan; 8 Department of Ecology and Evolution, University of Chicago, Chicago, IL 60637 USA

**Keywords:** Feather development, lncRNA, Zebra finch, ssRNA-seq

## Abstract

**Background:**

Long non-coding RNAs (lncRNAs) are important in various biological processes, but very few studies on lncRNA have been conducted in birds. To identify IncRNAs expressed during feather development, we analyzed single-stranded RNA-seq (ssRNA-seq) data from the anterior and posterior dorsal regions during zebra finch (*Taeniopygia guttata*) embryonic development. Using published transcriptomic data, we further analyzed the evolutionary conservation of IncRNAs in birds and amniotes.

**Results:**

A total of 1,081 lncRNAs, including 965 intergenic lncRNAs (lincRNAs), 59 intronic lncRNAs, and 57 antisense lncRNAs (lncNATs), were identified using our newly developed pipeline. These avian IncRNAs share similar characteristics with lncRNAs in mammals, such as shorter transcript length, lower exon number, lower average expression level and less sequence conservation than mRNAs. However, the proportion of lncRNAs overlapping with transposable elements in birds is much lower than that in mammals. We predicted the functions of IncRNAs based on the enriched functions of co-expressed protein-coding genes. Clusters of lncRNAs associated with natal down development were identified. The sequences and expression levels of candidate lncRNAs that shared conserved sequences among birds were validated by qPCR in both zebra finch and chicken. Finally, we identified three highly conserved lncRNAs that may be associated with natal down development.

**Conclusions:**

Our study provides the first systematical identification of avian lncRNAs using ssRNA-seq analysis and offers a resource of embryonically expressed lncRNAs in zebra finch. We also predicted the biological function of identified lncRNAs.

**Electronic supplementary material:**

The online version of this article (doi:10.1186/s12864-017-3506-z) contains supplementary material, which is available to authorized users.

## Background

A large portion of the eukaryotic genome is transcribed in the form of non-coding RNAs (ncRNAs) [1–3]. NcRNAs longer than 200 nucleotides are classified as long ncRNAs (lncRNAs), which are further divided into lincRNAs (long intergenic non-coding RNAs), intronic lncRNAs (transcribed within the introns of protein-coding genes), and lncNATs (long non-coding natural antisense transcripts, which are transcribed in the opposite strand of the protein-coding sequences) [4–7]. In general, lncRNAs show fewer exons, shorter transcript length and more diverse expression levels than protein-coding mRNAs [8, 9]. Furthermore, lncRNAs are usually evolutionarily less conserved in sequence than small/short ncRNAs and protein-coding mRNAs [8–10].

LncRNAs have been found to play regulatory and structural roles in diverse biological processes. For example, X-inactive specific transcript (*XIST*), a X-link lncRNA, mediates chromosome inactivation [11, 12], and *KCNQ1* overlapping transcript 1 (*KCNQ1OT1*), a paternally expressed lncRNA, regulates the establishment of genomic imprinting [13–15]. LncRNAs can work in *cis*- or *trans*-regulation. For example, *HOXA* transcript at the distal tip (*HOTTIP*) is the lncRNA produced from the 5' end of the *HOXA* locus that coordinates the activation of several 5' *HOXA* genes [16], while *HOX* transcription antisense RNA (*HOTAIR*) is the *trans*-acting lncRNA that is transcribed from the *HOXC* gene cluster but acts as the repressor on the *HOXD* gene cluster [17].

Mammal hair and avian feather have had evolved independently, but their developments share many signaling pathways [18, 19]. In hair formation, dermal papilla cells can be the source of dermal-derived signaling molecules and play crucial roles in hair follicle development and postnatal hair cycle. Several lncRNAs were predicted to interact with the Wnt signaling pathway during dermal papilla cell development [20]. Whether avian feather development is also regulated by lncRNAs is therefore an interesting question. A few studies on avian lncRNAs have been made [21–23] and Gardner et al. [21–23] have studied the conservation and losses of non-coding RNAs in avian genomes.

Natal down is the downy plumage in avian hatchlings. Natal down development starts with a series of reciprocal epithelio-mesenchymal molecular interactions between the dermis and the overlying epidermis to form the primordia. The signaling crosstalk between epidermis and dermis coordinates the spatial arrangement and regular outgrowth of feathers [24–26]. Our previous study investigated the natal down formation divergence in zebra finch (*Taeniopygia guttata*) hatchlings, using single stranded RNA-seq (ssRNA-seq) data from both the anterior and the posterior dorsal region of zebra finch embryos at developmental stages E8, E9 and E12 (Additional file 1: Figure S1) [27].

The purpose of this study was to identify lncRNAs in zebra finch, predict their function and study their evolutionary conservation in birds and amniotes. First, we designed a set of criteria to identified lncRNAs using the ssRNA-seq data of our previous study [27]. Second, we classified IncRNAs into lincRNAs, intronic lncRNAs and lncNATs and compared the genomic and expression features of the predicted lncRNAs with protein-coding genes and between zebra finch and mammals. Third, we predicted the functions of the IncRNAs in natal down development. Finally, we validated the expressions of candidate lncRNAs involved in natal down development by qPCR and studied the sequence conservation in amniotes.

## Results

### Identified lncRNAs

**Fig. 1:**
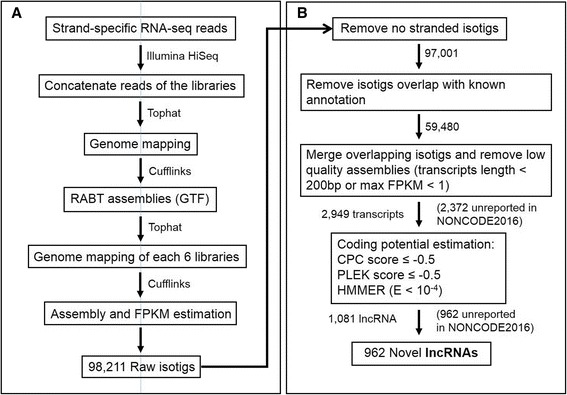
Overview of the ssRNA-seq transcriptome assembly and lncRNA identification pipeline. **a** Overview of the ssRNA-seq-based transcript reconstruction pipeline that was used to identify expressed transcripts in sequencing libraries. **b** The integrative pipeline for the stringent identification of lncRNAs in zebra finch dorsal skins. CPC: coding potential calculator; PLEK: predictor of long non-coding RNAs and messenger RNAs based on an improved k-mer scheme; HMMER (HMMER-3): Profiling protein sequence data using hidden Markov models

To identify lncRNAs in zebra finch, six ssRNA-seq datasets (E8A, E8P, E9A, E9P, E12A and E12P, Additional file 1: Figure S1 [26]) from anterior dorsal (AD) and posterior dorsal (PD) skins in three embryonic incubation days (E8, E9 and E12) were re-analyzed. To infer the consensus mapping locations of RNA-seq reads, the concatenated paired-end reads were aligned onto the zebra finch genome by TopHat and only properly paired reads were retained, resulting in the mapping rates of 77 to 79% for the libraries (Additional file 2: Table S1). The new annotation file (General Transfer Format, GTF file) generated by Cufflinks was used for the subsequently analyses (Fig. 1).

The strand specificities of the mapped reads were 86 to 92% for each library (Additional file 2: Table S1) [28], and the total number of the raw isotigs reconstructed using Cufflinks was 98,211 (Fig. 1). Raw isotigs without strand information (~1.3%) were removed and the remaining isotigs were separated to Ensembl annotated genes (Additional file 3: Table S2) and isotigs (59,480) that showed no overlap with any annotated genes (Fig. 1). We further merged the overlapping isotigs into raw transcripts (10,383). After removing the low quality assemblies as those with a small fragment (<200 bp) or low expression (max FPKM < 1 among all six libraries), we identified 2,949 unannotated transcripts, including 577 lncRNAs recorded in the NONCODE2016 database and 2,372 novel transcripts (Fig. 1; Additional file 4: Table S3) [29].

To identify lncRNAs, we focused on the unannotated transcripts. We first applied the coding potential calculator (CPC) to assess coding potential by considering the quality of predicted ORFs, and the homology with known proteins [30, 31]. In the 2,949 unannotated transcripts, 1,673 were identified as putative noncoding transcripts (Additional file 4: Table S3) by a cutoff score of −0.5 [8].

Although CPC has been widely used to analyze the coding potential, it only utilizes UniRef90 as the reference database [30, 32]. As the annotation of protein coding genes in the current bird genomes is not as complete as that in model mammals, it may include false positives in discovering lncRNAs. Our second approach was to use a newly developed classifier, known as the predictor of long non-coding RNAs and messenger RNAs based on an improved k-mer scheme (PLEK) [33] to estimate the coding potential of the transcripts, according to a training dataset generated from known coding and noncoding genes of chicken and zebra finch. We set the cutoff value to be −0.5 to reduce the possible bias in coding and noncoding gene classification. We identified 2,176 putative non-coding transcripts from the 2,949 unannotated transcripts (Additional file 4: Table S3).

The third approach was to eliminate the putative noncoding transcripts with similar reading frames with the Pfam protein domain database by HMMER3 (E-value < 10^−4^) [34]. Among the 2,949 unannotated transcripts, 2,265 transcripts remained and were taken as putative IncRNAs (Additional file 4: Table S3).

From the overlaps of the results of the three approaches, we identified 1,081 putative lncRNAs, including 119 lncRNAs annotated in NONCODE2016 [29] and 962 putative novel lncRNAs (Additional file 4: Table S3). The 1,081 lncRNAs could be classified into 965 lincRNAs, 59 intronic lncRNAs, and 57 lncNATs (Additional file 4: Table S3).

To evaluate our pipeline of coding potential estimation, we mapped the 1,081 putative lncRNAs and the remaining 1,868 unannotated transcripts to the zebra finch chromosomes (Additional file 5: Figure S2A). The 1,868 unannotated transcripts showed highest distribution in chromosome 25 and 27, while the 1,081 putative lncRNAs were distributed across all the chromosomes. Most α- and β- keratin genes were clustered in chromosomes 25 and 27 [35]. Keratin genes, especially β- keratin genes, are tandem duplicated genes with similar sequences. They are difficult to be annotated on the reference genome precisely and therefore many of them were included in our unannotated transcript pool. We mapped α- and β- keratin gene transcripts, unannotated transcripts (without lncRNAs), and lncRNAs to chromosomes 25 and 27 (Additional file 5: Figure S2B). In chromosome 25, the unannotated transcripts mainly overlapped with β- keratin genes, while in chromosome 27, the unannotated transcripts mainly overlapped with α- keratin genes. However, the overlap between lncRNAs and keratin genes was lower than that between unannotated transcripts and keratin genes (Additional file 5: Figure S2A), suggesting that our pipeline for lncRNAs identification could effectively exclude keratin-like transcripts. Chromosomes 25 is short (Chr. 25: 1.28 Mb; Chr. 26: 4.91 Mb; Chr. 27: 4.62 Mb) and therefore the values of “Transcript number/Chromosome size (Mb)” are very high for Chr. 25 (Additional file 5: Figure S2A).

The distribution range of the putative lncRNAs is from 0.40 to 3.91 lncRNAs per chromosome. We mapped the previous identified lncRNAs expressed in human skin to human chromosomes (except the Y chromosome) and found that the distribution range of the lncRNAs across the chromosomes is from 0.56 to 2.99 lncRNAs per chromosome [36], which is close to the distribution range of zebra finch skin lncRNAs we identified.

### Genomic and expression features of the putative lncRNAs

**Fig. 2:**
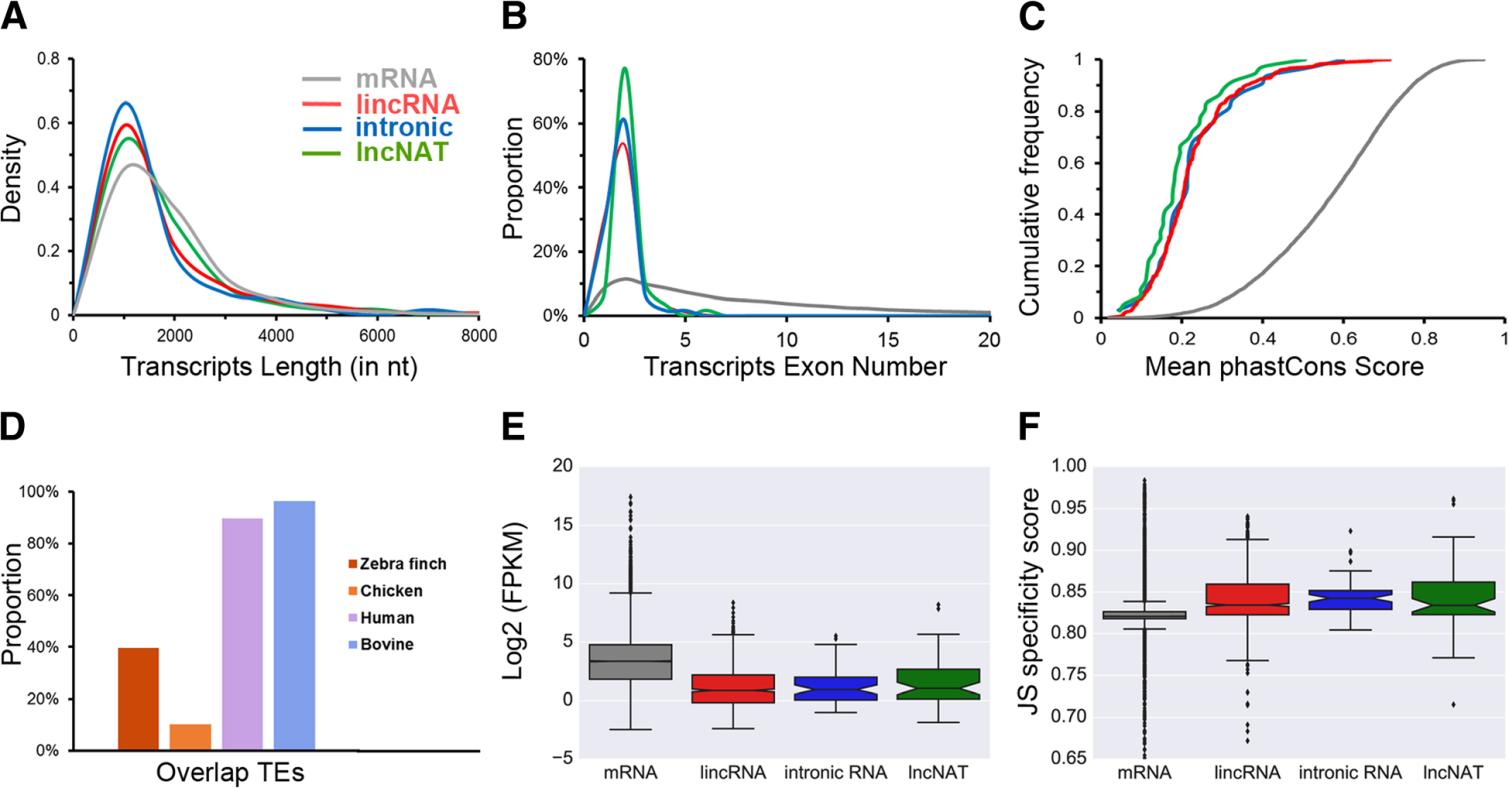
Genomic and expression features of the predicted zebra finch lncRNAs. Genomic features of (**a**) transcripts length, (**b**) exon number, and (**c**) mean phastCons score of zebra finch mRNA, lincRNA, intronic lncRNA, and lncNAT were compared. **d** The fraction of lncRNAs overlapping with at least one base of a TE (transposable element) in zebra finch, chicken, human, and bovine. Expression features of (**e**) expression levels and (**f**) JS scores of zebra finch mRNA, lincRNA, intronic lncRNA, and lncNAT were compared

We compared the transcript lengths, exon counts and sequence conservation of the 1081 putative lncRNAs with the protein-coding mRNAs. In agreement with previous studies in mammals [6, 8, 36, 37], the length distribution of the identified lncRNAs (median 0.75 kb; average 1.32 kb) is shorter than that of the mRNAs (median 1.09 kb; average 1.47 kb; *p* < 10^−8^, Student’s *t*-test), while the length distribution shows no significant differences between lincRNA, intronic lncRNA, and lncNAT (Fig. 2a). The exon counts of the putative lncRNAs (average 1.9 exons per transcript) is also less than that of the mRNAs (average 10.3 exons per transcript; *p* < 0.0001, Student’s *t*-test), while the exon counts of the three kinds of lncRNA show no differences (Fig. 2b). The sequences are less evolutionarily conserved in the putative lncRNAs than in protein-coding mRNAs (Fig. 2c). Finally, the proportions of overlapping lncRNAs and TEs in birds (zebra finch 39.6%; Chicken 10.3%) are much lower than those in mammals ((human 89.8%; bovine 96.4%, Fig. 2d; Additional file 6: Table S4), suggesting that TEs are not a major origin of avian lncRNAs.

We also compared the expression levels and the tissue specificities of the putative lncRNAs with those of the protein-coding mRNAs. The average expression levels of the putative lncRNAs (median 1.7; average 6.3 FPKM) tend to be lower than those of the mRNAs (median 9.6; average 114.7 FPKM; *p* < 0.0001, Student’s *t*-test; Fig. 2e). To quantify the tissue specificity of the transcripts of mRNA, lincRNA, intronic lncRNA, and lncNAT, we compared the JS scores [38] of the expressed transcripts between different skin regions and between different developmental stages. The results showed that the regional specificity is significantly different between the mRNAs and the lncRNAs (*p* < 0.0001, Student’s *t*-test; Fig. 2f), but no significant difference could be detected between different types of lncRNAs. Furthermore, no significant difference was detected between different types of lncRNAs in the three developmental stages analyzed (Additional file 7: Figure S3; also see Methods of [26]).

### Co-expression analysis

**Fig. 3:**
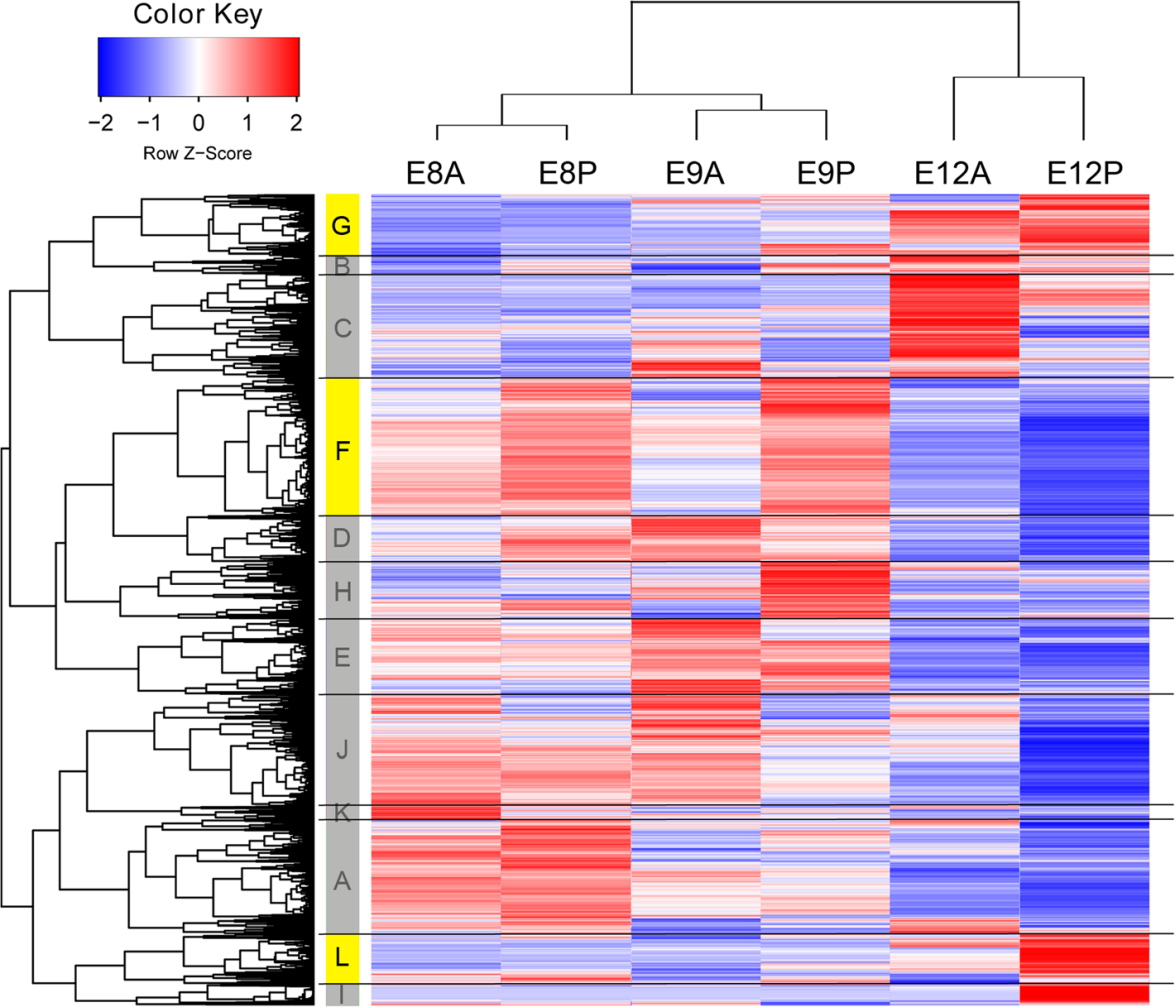
Clustering analysis of the expressed genes and the expression heat map. Hierarchical clustering analysis clustered the 13,362 expressed annotated genes and 2,949 unannotated transcripts into 12 clusters (A-L, see Additional files tables for details). The expression levels of each gene are shown as the scaled FPKM values across the six transcriptomes (scaled z-score: red = up-regulation, blue = down-regulation). Three clades (F, G, and L) used for further analysis were labeled in yellow

Most lncRNAs lack annotated features and functional predictions for the lncRNAs have often been based on “guilt-by-association” analysis [38–40]. We clustered the lncRNAs along with the Ensembl functional annotated genes according to their expression profiles, and analyzed the GO categories enriched in each cluster. The expressed genes were classified into 12 expression clusters (A-L) (Fig. 3; Additional file 3: Table S2 and Additional file 4: Table S3). Then, we utilized the website based software g: Profiler to analyze the gene set enrichment of each cluster and excluded the clusters that may not be associated with natal down development by a series of filters; the detail of the filtering is described in Additional file 8: Supplementary Results. Only Clusters F, G, and L passed our criteria and were potentially associated with feather formation. To confirm the functional categories of these clusters, we further conducted Fisher’s exact test to gain the enrichments of GO terms and protein domains (collected from zebra finch protein domain databases: Pfam, Interpro, SMART, and SUPERFAMILY) in the three clusters. Only the GO categories with a *p* value < 0.01 and FDR < 0.05 were analyzed further.

Genes in Cluster F were enriched in transcription factors (PF00076), mRNA metabolic process (GO:0016071), cell cycle process (GO:0022402), and DNA replication (GO:0006260) (Additional file 9: Table S5, Additional file 10: Table S6 and Additional file 11: Table S7), suggesting that lncRNAs in this cluster may be associated with cell proliferation. A previously identified feather bud growth promoter, *sonic hedgehog* (*SHH*), was in this cluster and expressed higher in downy dorsal skin than in naked dorsal skin [27]. Genes in Cluster G were enriched in the Claudin family (PF00822), the Rho protein signaling pathway (GO:0051056, GO:0046578, and PF00621), skin development (GO:0043588), keratinocyte differentiation (GO:0030216), and epithelial cell differentiation (GO:0030855) (Additional file 9: Table S5, Additional file 10: Table S6 and Additional file 11: Table S7). Claudins are the main component of tight junctions and Rho family GTPases are known to regulate the tight junctions [41]. A previous study showed that tight junctions are associated with the formation of feather branches, suggesting that lncRNAs in this cluster may regulate feather morphogenesis [42]. In Cluster L, genes showed enrichment in α-keratin domain (intermediate filament protein, PF00038) (Additional file 9: Table S5, Additional file 10: Table S6 and Additional file 11: Table S7). Although the FDR value of the protein domain enrichment exceeded 0.05, we still considered this result significant because α- keratin domains were trained based on mammalian data, so the calculation of FDR in avian α- keratin domains might be overestimated. [35]. Several β-keratins were also clustered in this cluster (Additional file 3: Table S2). It is possible that the lncRNAs in this cluster are involved in feather formation.

### Validation and sequence analysis of the candidate lncRNAs associated with natal down development

To find the lncRNAs associated with natal down development in birds, we focused only on the lncRNAs that satisfied the following criteria: First, the lncRNAs were clustered in Cluster F, G, or L. Second, the lncRNAs were differentially expressed between the AD and PD skin regions (Additional file 4: Table S3). Third, the lncRNAs shared similar sequences in the same chromosomes between zebra finch and chicken. Three candidate lncRNAs, CUFF.19772.1 (in Cluster F), CUFF.6222.3 (in Cluster G), and CUFF.14902.2 (in Cluster L), were selected for further analysis. The sequence of CUFF.19772.1 is recorded in the NONCODE lncRNA database (ID: NONBTAT021324 and NONMMUT059481, found in bovine and mouse, respectively). CUFF.6222.3 and CUFF.14902.2 were putative novel lncRNAs.

**Fig. 4:**
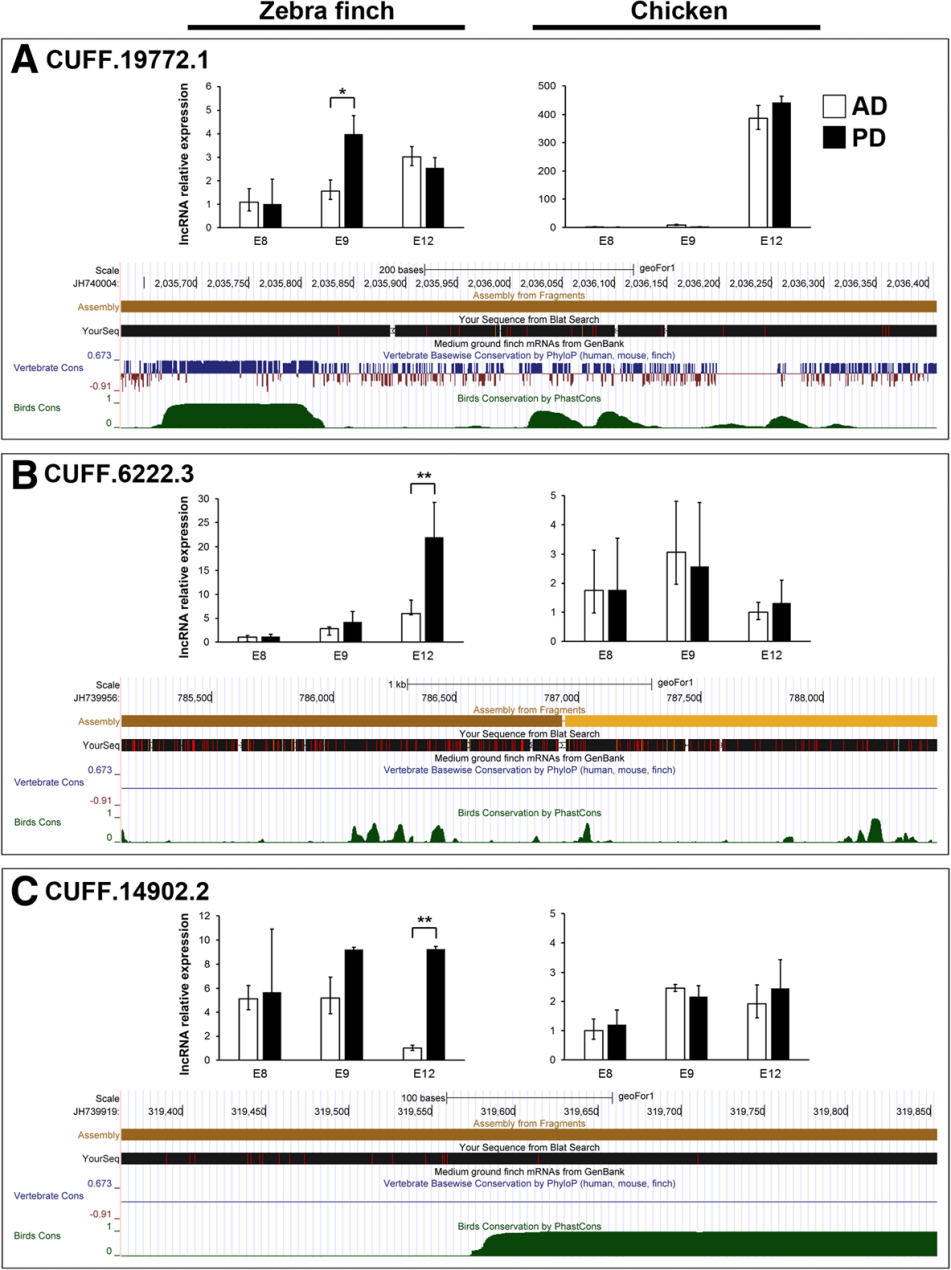
Quantitative PCR and sequence conservation analysis of the three identified lncRNAs. **a** Expression profiles of lncRNA CUFF.19772.1 in E8, E9, and E12 of zebra finch and chicken AD and PD skins. **b** Expression profiles of lncRNA CUFF.6222.3 in E8, E9, and E12 of zebra finch and chicken AD and PD skins. **c** Expression profiles of lncRNA CUFF.14902.2 in E8, E9, and E12 of zebra finch and chicken AD and PD skins. Sequence conservations are shown below the quantitative PCR in blue (conservation among amniotes) and green (conservation among birds)

The expression levels of the predicted lncRNAs were too low to be detected by whole mount in situ hybridization. To confirm the role of the three selected putative lncRNAs, we compared their expression levels in the AD and PD skins of different individuals of zebra finch and chicken by quantitative PCR. All three lncRNAs were expressed in both zebra finch and chicken. Moreover, in zebra finch, those lncRNAs were expressed more highly in the PD region than in the AD region, but no expression differences could be detected between the AD and PD skin regions in chicken (Fig. 4). Zebra finch has two types of natal down formation in dorsal skins, but chicken only has one type (Additional file 1: Figure S1). Our previous study had found that most feather formation genes were differentially expressed between the AD and PD skin regions in zebra finch, but not in chicken [27]. Therefore, these three lncRNAs might be involved in natal down development.

We studied the sequence conservation of these three lncRNAs between birds and between amniotes. The multiple genome alignment of the medium ground finch in the UCSC Genome Browser provided the sequence conservation scores across birds (zebra finch, chicken, turkey, and budgerigar) and across amniotes (birds, human, and mouse) [43]. We used the UCSC BLAT algorithm to map our lncRNA sequences to the genome of medium ground finch to evaluate the sequence conservation (Fig. 4). In CUFF.19772.1, the sequence was conserved in both birds and amniotes (Fig. 4a), suggesting a function shared by amniotes. In CUFF.6222.3, the sequence has been only partially conserved in birds (Fig. 4b). In CUFF.14902.2, the sequence has been highly conserved only in birds (Fig. 4c). Interestingly, we found that CUFF.19772.1 is similar in sequence with the 3’ UTR of human *BHLHE41* (the basic helix-loop-helix family, member e41, Additional file 12: Figure S4). BHLHE41 is a transcription factor and known to be the upstream signal of c-Myc [44], and c-Myc could promote the epithelium cell proliferation in feather bud elongation [45]. In our transcriptomes, the expression profiles of *BHLHE41* and *MYC* belong to the same cluster with CUFF.19772.1 (Cluster F, Additional file 3: Table S2). Taken together, these results suggest that through the c-Myc signaling, CUFF.19772.1 promotes feather bud elongation.

## Discussion

In this study, we developed a pipeline to identify zebra finch lncRNAs from the published ssRNA-seq data. We analyzed the genomic and expression features of the identified lncRNAs and compared the features with that in other vertebrates. We constructed a weighted gene co-expression network and predicted the functions of the lncRNAs based on their correlation with known protein-coding genes.

To find candidate lncRNAs in natal down formation, we compared the zebra finch lncRNA from AD and PD skins. Then, we compared the expression profiles of the candidate lncRNAs in zebra finch with those in chicken to identify avian conserved lncRNAs, which may be involved in natal down development. Feathers play important roles in heat conservation, mate attraction, physical protection, and flight. Many signaling molecules of these processes are well established in chicken [45–52]. However, as most previous studies focused on protein-coding genes, the role of non-coding RNAs (ncRNAs) in feather development is unclear.

In agreement with the previous studies in various eukaryotes [6–8, 53], our identified lncRNAs have shorter transcript length, lower exon number, lower sequence conservation, less average expression, and higher tissue specific expression than protein-coding transcripts. However, we found the overlapping proportions between lncRNAs and TEs are much lower in birds than in mammals. Previous studies proposed that TEs are one of the major origins of lncRNAs in vertebrates, and TEs embedded in lncRNAs are subjected to RNA editing or secondary structure formation [54, 55]. However, these studies did not include avian lncRNAs. Birds are known to have lower percentages of TEs in their genomes than most other vertebrates [56]. Thus, it seems that TEs have a lower contribution to lncRNAs in birds than in mammals. Although several lncRNAs play an essential role in cellular differentiation, cell lineage choice, organogenesis and tissue homeostasis, the function of most identified lncRNAs is unknown [57]. In our tissue specificity analysis, we found differential expression of lncRNAs among skin regions but not among developmental stages. Thus, our identified lncRNAs may play a role in skin or skin appendage differentiation, although probably not in skin or skin appendage growth.

In general, most lncRNAs show low primary sequence conservation between species despite having similar functions. In our study, one putative natal down development associated lncRNAs showed sequence conservation among amniotes. This is an interesting observation because feather and hair share many molecules at the start of their development, although hair and feather utilize different molecules for morphogenesis and cornification. LncRNA CUFF.19772.1 showed high sequence conservation among human, mouse, and birds. Moreover, the co-expressed *SHH* and *MYC* are important molecules that promote cell proliferations for both feather and hair formation [58–60]. Although the function of the host gene *BHLHE41* in hair formation is not known, we speculate that CUFF.19772.1 is important for early stages of both feather and hair formation. Through c-Myc signaling, CUFF.19772.1 might interact with or function like *SHH* to promote feather bud elongation [27, 60]. In contrast, lncRNA CUFF.6222.3 and CUFF.14902.2 are co-expressed with feather morphogenesis and cornification factors, such as Claudins, Rho proteins, and α- and β-keratins, and their sequences have been conserved only in birds. CUFF.14902.2 showed high sequence conservation in birds and is located in chromosome 17. Most feather cornification factors, such as α- and β-keratins, are not located in chromosome 17, but are clustered in chromosomes 2, 25, 27, and 33 in both zebra finch and chicken [35, 61]. Therefore, we propose that CUFF.14902.2 may be associated with feather cornification in trans-regulation. Furthermore, all the three conserved lncRNAs we found do not overlap with any of the previously identified well conserved lncRNAs [23].

Several concerns arise from the analysis of this study. First, previous pipelines for lncRNA predictions in mammals excluded single-exon transcripts [19, 21]. However, compared to mammals, bird genomes are more compact with shorter introns and intergenic regions [22, 62, 63]. Therefore, we retained single exon transcripts in our lncRNA pool. Second, we used zebra finch as the model animal in this study because its unique natal down growth feature enabled us to find candidate regulators for natal down formation. However, the average protein-coding transcript length is much longer in chicken (2.3 kb) than that in zebra finch (1.47 kb), and as 1/6 of the sequences are unassigned to chromosomes, the assembly quality of the zebra finch genome is not as good as those of other model animals, and so some lncRNAs may have been missed in our data. The fast growing avian genome sequencing data may help to remove these concerns in the future [22].

## Conclusion

Previous lncRNA studies covered many organisms, but less include birds. In this study, we employed ssRNA-seq to identify zebra finch lncRNAs and predicted the function of the identified lncRNAs. We identified 962 novel lncRNAs, which greatly expanded the repertoire of lncRNAs. In genomic feature analysis of the identified lncRNAs, we found that TEs are not a major origin of avian lncRNAs. Moreover, by comparing the expression profiles between zebra finch and chicken, and by examining the sequence conservation among amniotes, three lncRNAs were found to have been highly conserved and were predicted to be associated with natal down development.

## Methods

### RNA isolation

The zebra finch and chicken embryonic skin tissues were dissected as described in Additional file 1: Figure S1 (red dash boxes, AD: anterior dorsal skin; PD: posterior dorsal skin). Tissue total RNA was isolated and quality assessed as described in Chen et al. [27].

### Data processing, reads mapping and assembly

Sequencing reads of the six libraries were described in Chen et al. [27] and summarized in Additional file 1: Figure S1 and Additional file 2: Table S1. This study used the new versions of Tophat (version 2.0.14) and Cufflinks (version 2.2.1) to process the reads. The zebra finch genome (version Taeniopygia_guttata.taeGut3.2.4) and its gene annotation were downloaded from Ensembl. The processed sequencing reads were then mapped to the genome using Tophat [64], and its embedded aligner Bowtie (version 2.1.0) [65] by the following parameters: −r 116 --mate-std-dev 100 --library-type fr-firststrand -g 2. The normalized expression levels of genes, represented by fragments per kilobase of exon per million fragments mapped (FPKMs) [66], were generated by Cufflinks [67] by the following parameters: −−library-type fr-firststrand --max-bundle-frags 10^12^.

### Identification of novel transcripts

The pipeline for exploring novel transcripts is shown in Fig. 1. Raw transcripts generated from our mapping and assembly were filtered by the following criteria to detect putative novel transcripts: 1. Transcripts that have no strand information were removed. 2. Transcripts that overlap with the locations of the annotated genes in the Ensemble and UCSC databases were removed. 3. Transcripts with length less than 200 bp or an FPKM value lower than 1 in all the libraries were removed. 4. Transcripts not recorded in the NONCODE2016 database were retained [29].

### Coding potential analysis

The coding potential calculator (CPC) is a SVM-based classifier based on the presence and integrity of the ORF in a transcript and on the Blastx-computed similarity scores between transcript ORFs and the known protein databases [30, 31]. UniRef90 [32] was used as the protein reference for the analysis and we set the cutoff score of −0.5 to distinguish noncoding RNAs from coding RNAs.

The predictor of long non-coding RNAs and messenger RNAs based on an improved k-mer scheme (PLEK) is a newly developed classifier based on the improved *k*-mer scheme and a SVM algorithm [33]. We used Ensembl known coding-genes of zebra finch (Taeniopygia_guttata.taeGut3.2.4.cds.all.fa) and known noncoding genes from the combination of chicken and zebra finch (Taeniopygia_guttata.taeGut3.2.4.ncrna.fa and Gallus_gallus.Galgal4.ncrna.fa) as the training dataset to score the novel transcripts. We stringently set the cutoff value to be −0.5 for the coding and noncoding genes discrimination.

### Genomic and expression features of the identified lncRNAs

We analyzed several commonly characterized genomic and expression features of the identified lncRNAs according to the previous studies [6, 8, 36]. The identified 1,081 lncRNAs and the 16,869 protein-coding mRNA were used in the analysis (Additional file 3: Table S2; Additional file 4: Table S3).

#### Conservation analysis

We generated the three birds multiple genome alignment. Zebra finch (Taeniopygia_guttata.taeGut3.2.4) was used as the target, and chicken (Gallus_gallus.Galgal4) and flycatcher (Ficedula_albicollis.FicAlb_1.4) were used as the queries. Briefly, we downloaded the homologous genes between the species from the Ensembl database. These homologous genes were used as the anchors to construct the multi-species genomic synteny blocks. These syntenic blocks were aligned by Multiz-TBA (threaded blockset aligner) software to generate three species multiple genome alignment [68]. The average phastCon score of the location of the predicted lncRNAs and protein-coding genes were calculated by phastCons software [69]. Nucleotides which have no phastCon score were ignored.

#### Transposable element overlapping analysis

We analyzed the TEs and lncRNAs of human, bovine, zebra finch, and chicken. The locations of SINE, LINE, LTR, and DNA transposable elements generated by RepeatMasker were downloaded from the UCSC table browser. To reduce the possible bias from the tissue specificity of the lncRNAs, we collected published lncRNAs from similar tissues in different species. The genome version and the lncRNAs datasets were based on the previous studies in human skin [36], bovine muscle [6], and chicken muscle [21] (Additional file 6: Table S4).

#### Evaluation of tissue specificity

We estimated the tissue specificity of an expressed gene based on the JS (Jensen-Shannon) score. A higher JS score indicates a higher degree of tissue specific expression under that condition. We used the maximum JS score among the libraries of a transcript to represent the expression specificity of the transcript. Regional and developmental stage specificities are the two conditions used in our analysis.

### Clustering analysis and differentially expressed genes (DEGs) identification

In the clustering analysis, we first defined an expressed gene as having a FPKM value > 1 in at least one library. All the expressed known genes and the identified 2,949 transcripts (1,868 unannotated protein-coding transcripts and 1,081 lncRNAs) were hierarchically clustered by the WPGMA (Weighted Pair-Group Method with Arithmetic mean) method by the R script. Heatmap of the clusters was generated by Heatmap.2. The cut-off for the cluster analysis was 0.69.

We identified the DEGs (differentially expressed genes) through several sets of comparisons. To identify the candidate genes (protein-coding gene and lncRNAs) involved in natal down developments, we compared the regional gene expression differences between the AD and PD skin regions in the three embryonic incubation days. To increase the power of detecting the DEGs with low expression, the libraries of AD skins were used as the AD replicate, while the libraries of PD skins were used as the PD replicate. The two replicates were further compared (E8A + E9A versus E8P + E9P, and E9A + E12A versus E9P + E12P). To identify the candidate genes (protein-coding gene and lncRNAs) for skin development, we compared the temporal gene expression differences between different embryonic incubation days in AD or PD skin regions. The DEGs from the comparisons were estimated by NOISeq [70]. Only the genes with q > 0.7 were defined as differentially expressed [71]. All DEGs were labeled in Additional file 4: Table S3.

### Gene set enrichment and pathway analysis

To search the possible pathways involved in natal down development, the Ensemble gene ID of the expressed genes were converted to the ID of their chicken homologs and input into g:Profiler, a web-based toolset for functional profiling of gene lists from large-scale experiments. The p-value of the gene enrichment was corrected by Benjamini-Hochberg FDR (false discovery rate). Only the gene ontology with the corrected *p*-value < 0.05 was used in further analyses.

### Quantitative PCR

To quantify the candidate lncRNA gene expression levels, the cDNAs were synthesized from the total RNAs by QuaniTect Reverse Transcription kit (Qiagen). Each cDNA sample containing SYBR green (KAPA SYBR FAST qPCR kit) was run on LightCycler 480 (Roche) under the appropriate conditions. Quantification of the TATA box binding protein (TBP) RNA was used to normalize target gene expression levels. All the PCR primers are listed in Additional file 13: Table S8.

## Additional files

Additional file 1: Figure S1. Schematic presentation of natal down development in zebra finch and chicken and the six ssRNA-seq datasets used in this study. (A) Zebra finch embryos show two types of feather formation. The anterior dorsal (AD) tract and its two flanks show Type I feather formation (open circles) in which the feather buds do not develop into feather. On the other hand, the middle stripe of the posterior dorsal (PD) tract and the other regions shown in black circles show Type II feather formation in which the feather buds develop into down feathers, which are later replaced by contour feathers. In contrast, both the AD and the PD region of chicken embryos show Type II feather formation. (B) The skin regions and the six ssRNA-seq datasets used in this study. To reduce the complexity of skin regional specificity, only the dorsal skins (red dash boxes) were dissected and analyzed for the gene expressions. E8A: AD skin of embryo day 8; E8P: PD skin of embryo day 8; E9A: AD skin of embryo day 9; E9P: PD skin of embryo day 9; E12A: AD skin of embryo day 12; E12P: PD skin of embryo day 12; D7: 7 days post-hatch. Scale bar: 0.1 cm. The figure was modified from our previous study [27]. (TIF 20328 kb)
Additional file 2: Table S1. Read count statistics of the ssRNA seq. (DOCX 13 kb)
Additional file 3: Table S2. Information of the annotated genes. (XLSX 1440 kb)
Additional file 4: Table S3. 2,949 identified transcripts information. (XLSX 517 kb)
Additional file 5: Figure S2. Chromosomal distribution of the expressed unannotated transcripts. (A) Chromosomal distribution of the expressed 2949 transcripts and 1080 lncRNAs. The Y axis represents the transcript number per million bases of the chromosome. (B) The distributions of α- keratin gene transcripts, β- keratin gene transcripts, unannotated transcripts (without lncRNAs), and lncRNAs in chromosomes 25 and 27. Purple line: α- keratin gene transcripts; green line: β- keratin gene transcripts; orange line: unannotated transcripts (without lncRNAs); blue line: lncRNAs. *P*-value < 0.05 is indicated by dashed red line (chi-square test with one-tailed). (TIF 17522 kb)
Additional file 6: Table S4. Tables of the collected human, chicken, bovine lncRNAs. (XLSX 90 kb)
Additional file 7: Figure S3. The distribution of Maximal JS stage specificity score. The JS score distributions for mRNA (grey box), lincRNA (red box), intronic lncRNA (blue box), and lncNAT (green box). A higher score value indicates a higher degree of stage specificity expression. (TIF 145 kb)
Additional file 8: Supplementary Results. (DOCX 22 kb)
Additional file 9: Table S5. Gene set enrichment analysis using gProfiler. (XLSX 743 kb)
Additional file 10: Table S6. Gene set enrichment analysis using Fisher’s exact test. (XLSX 1040 kb)
Additional file 11: Table S7. Protein domains enriched in clades F, G and L. (XLSX 442 kb)
Additional file 12: Figure S4. The location of the aligned CUFF.19772.1 (black bar) and *BHLHE41* in human genome. CUFF.19772.1 was aligned to the 3’ UTR of human gene *BHLHE41*. (TIF 234 kb)
Additional file 13: Table S8. Primer pair sequences used in this study. (DOCX 15 kb)

## Abbreviations

AD: Anterior dorsal skin region
E12A: AD skin of embryo day 12
E12P: PD skin of embryo day 12
E8A: AD skin of embryo day 8
E8P: PD skin of embryo day 8
E9A: AD skin of embryo day 9
E9P: PD skin of embryo day 9
PD: Posterior dorsal skin region
ssRNA-seq: Single-stranded RNA-seq
