# Supplementary figures and images for: Identification and evolutionary analysis of long non-coding RNAs in zebra finch
Source: BMC Genomics. 2017 Jan 31;18:117. doi: 10.1186/s12864-017-3506-z (PMC5282891; doi:10.1186/s12864-017-3506-z)

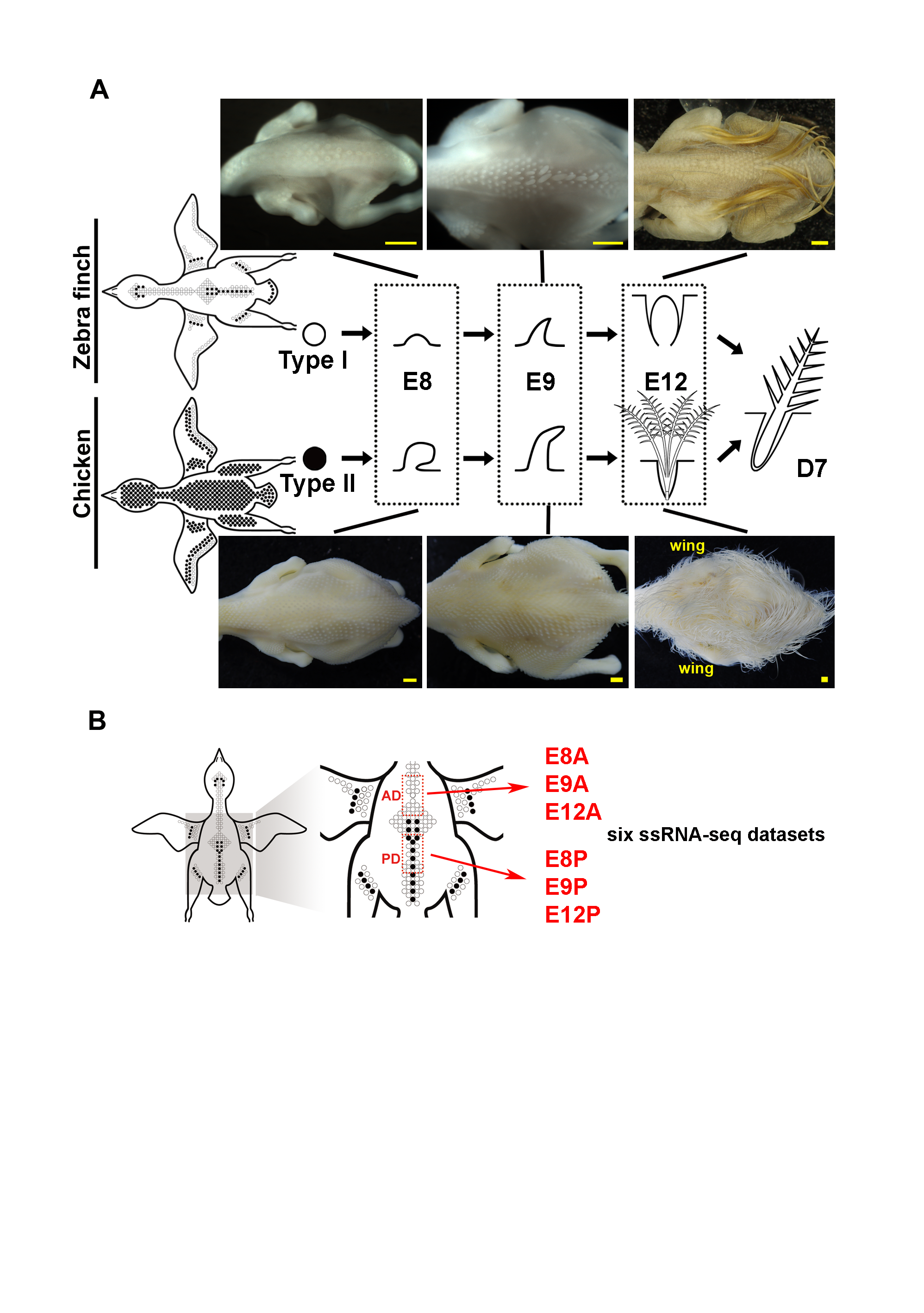

Supplement: Additional file 1: Figure S1. — Schematic presentation of natal down development in zebra finch and chicken and the six ssRNA-seq datasets used in this study. (A) Zebra finch embryos show two types of feather formation. The anterior dorsal (AD) tract and its two flanks show Type I feather formation (open circles) in which the feather buds do not develop into feather. On the other hand, the middle stripe of the posterior dorsal (PD) tract and the other regions shown in black circles show Type II feather formation in which the feather buds develop into down feathers, which are later replaced by contour feathers. In contrast, both the AD and the PD region of chicken embryos show Type II feather formation. (B) The skin regions and the six ssRNA-seq datasets used in this study. To reduce the complexity of skin regional specificity, only the dorsal skins (red dash boxes) were dissected and analyzed for the gene expressions. E8A: AD skin of embryo day 8; E8P: PD skin of embryo day 8; E9A: AD skin of embryo day 9; E9P: PD skin of embryo day 9; E12A: AD skin of embryo day 12; E12P: PD skin of embryo day 12; D7: 7 days post-hatch. Scale bar: 0.1 cm. The figure was modified from our previous study [27]. (TIF 20328 kb) [file 12864_2017_3506_MOESM1_ESM.tif]

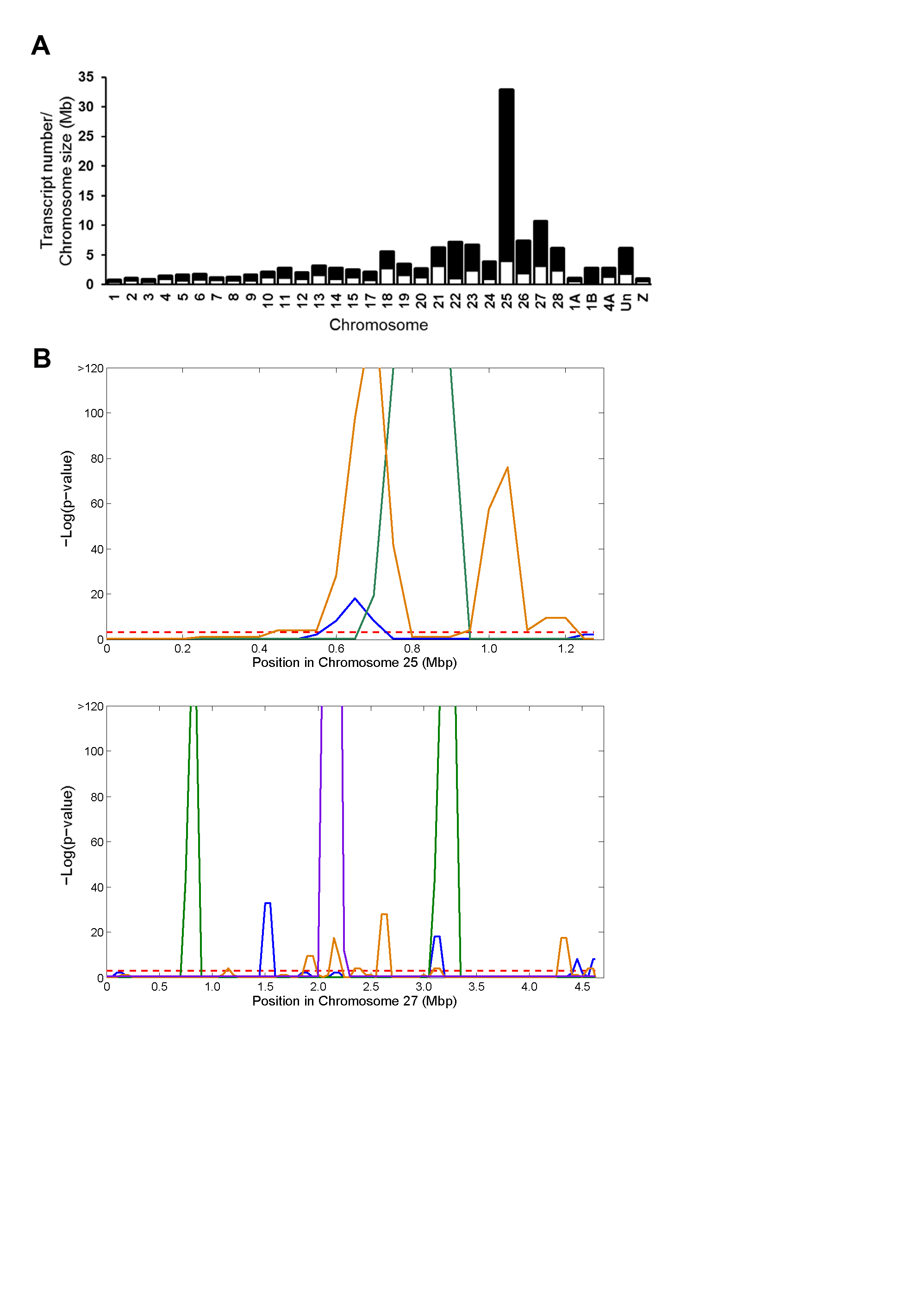

Supplement: Additional file 5: Figure S2. — Chromosomal distribution of the expressed unannotated transcripts. (A) Chromosomal distribution of the expressed 2949 transcripts and 1080 lncRNAs. The Y axis represents the transcript number per million bases of the chromosome. (B) The distributions of α- keratin gene transcripts, β- keratin gene transcripts, unannotated transcripts (without lncRNAs), and lncRNAs in chromosomes 25 and 27. Purple line: α- keratin gene transcripts; green line: β- keratin gene transcripts; orange line: unannotated transcripts (without lncRNAs); blue line: lncRNAs. P-value < 0.05 is indicated by dashed red line (chi-square test with one-tailed). (TIF 17522 kb) [file 12864_2017_3506_MOESM5_ESM.tif]

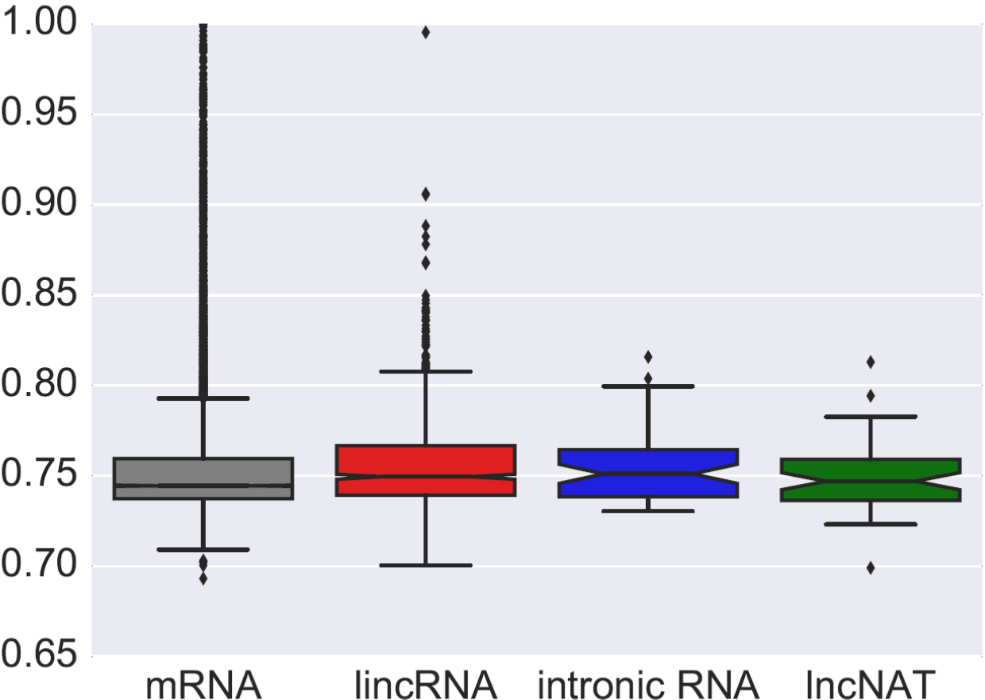

Supplement: Additional file 7: Figure S3. — The distribution of Maximal JS stage specificity score. The JS score distributions for mRNA (grey box), lincRNA (red box), intronic lncRNA (blue box), and lncNAT (green box). A higher score value indicates a higher degree of stage specificity expression. (TIF 145 kb) [file 12864_2017_3506_MOESM7_ESM.tif]

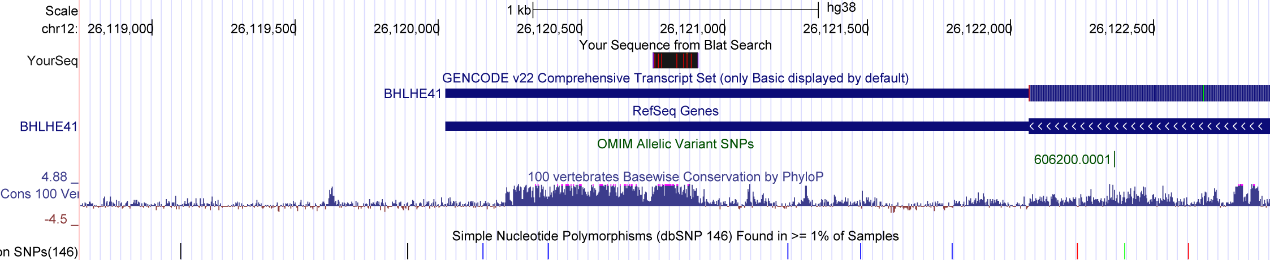

Supplement: Additional file 12: Figure S4. — The location of the aligned CUFF.19772.1 (black bar) and BHLHE41 in human genome. CUFF.19772.1 was aligned to the 3’ UTR of human gene BHLHE41. (TIF 234 kb) [file 12864_2017_3506_MOESM12_ESM.tif]
