# Supplementary material for: Identification and evolutionary analysis of long non-coding RNAs in zebra finch
Source: BMC Genomics. 2017 Jan 31;18:117. doi: 10.1186/s12864-017-3506-z (PMC5282891; doi:10.1186/s12864-017-3506-z)
Supplement: Additional file 2: Table S1. — Read count statistics of the ssRNA seq. (DOCX 13 kb) [file 12864_2017_3506_MOESM2_ESM.docx]

**Table S1. Read count statistics of the strand-specific Illumina deep RNA sequencing data for the 6 libraries studied.**

|  |  | Quality filtering | |  | Mappable reads | |  | Strand specificity |
| --- | --- | --- | --- | --- | --- | --- | --- | --- |
| Library | Type | Total reads | Rate (%) |  | Filtered reads | Rate (%) |  | Rate (%) |
| E8A^a^ | 101nt, PE^b^ | 212,574,848 | 90.0 |  | 192,135,468 | 77.2 |  | 89.56 |
| E8P^a^ | 101nt, PE^b^ | 195,347,384 | 90.9 |  | 177,704,990 | 78.2 |  | 89.51 |
| E9A^a^ | 101nt, PE^b^ | 209,108,402 | 89.9 |  | 187,884,810 | 76.3 |  | 88.87 |
| E9P^a^ | 101nt, PE^b^ | 243,286,006 | 90.2 |  | 219,415,584 | 79.3 |  | 86.87 |
| E12A^a^ | 101nt, PE^b^ | 238,270,682 | 90.0 |  | 214,572,150 | 77.2 |  | 89.15 |
| E12P^a^ | 101nt, PE^b^ | 179,208,508 | 89.9 |  | 161,249,104 | 78.2 |  | 92.84 |

^a^E8A: anterior dorsal skin in 8th embryonic incubation days; E8P: posterior dorsal skin in 8th embryonic incubation days; E9A: Anterior dorsal skin in 9th embryonic incubation days; E9P: posterior dorsal skin in 9th embryonic incubation days; E12A: Anterior dorsal skin in 12th embryonic incubation days; E12P: posterior dorsal skin in 12th embryonic incubation days

^b^PE: paired-end
