# Supplementary material for: Identification and evolutionary analysis of long non-coding RNAs in zebra finch
Source: BMC Genomics. 2017 Jan 31;18:117. doi: 10.1186/s12864-017-3506-z (PMC5282891; doi:10.1186/s12864-017-3506-z)
Supplement: Additional file 8: — Supplementary Results. (DOCX 22 kb) [file 12864_2017_3506_MOESM8_ESM.docx]

**Supplementary Results**

We listed the number of the expressed genes, unannotated transcripts, lncRNAs, and DEGs (differential expressed genes) from the six transcriptomes in the table below. The proportions of the expressed unannotated transcripts, lncRNAs, and DEGs in the expressed genes were also calculated and shown in the brackets. To narrow down the targets of the functional groups, we only focused on the clades that include more than 1000 genes and 10 % DEGs. Clades C, F, G, H, J and L were selected and further analyzed by gene enrichment analysis tool g:Profiler [[1](#_ENREF_1)].

Clade C includes more than 2,000 genes and has highest number and proportion of lncRNAs. However, GO categories were enriched in muscle development or structure (GO:0030016, GO:0030017, GO:0043292, and GO:0003012; Additional file 8: Table S5). Our previous study had shown that muscle tissues are easier to adhere to the anterior dorsal skin than the posterior dorsal skin. Therefore, these GO categories and DEGs should be the bias from the sample collection [[2](#_ENREF_2)].

The GO categories in Clade H were enriched in cellar component (GO:0043227, GO:0005737, GO:0044428 and GO:0044446; Additional file 8: Table S5). These results might be due to the fact that cell mass is larger in posterior dorsal skin and the above natal down than in anterior dorsal skin. However, we cannot find appropriate lncRNAs in this clade for further analysis.

The GO categories in Clade J were enriched in anatomical structure (GO:0048856, GO:0032502, GO:0007275, and GO:0048731; Additional file 8: Table S5). Many differentially expressed *HOX* genes are in this clade. However, our previous study showed that the enrichment of this group of genes come from the skin regional specificity, but not from the natal down developmental differences [[2](#_ENREF_2)].

Therefore, in this study, we focused on the lncRNAs in Clades G, F, and L.

**The clusters and the expressed gene, lncRNA, DEGs number in each cluster**

| Clade | | Expressed Genes | Unannotated transcripts | | lncRNAs | | DEGs | |
| --- | --- | --- | --- | --- | --- | --- | --- | --- |
| A | 2,298 | | | 424 (18 %) | | 162 (7 %) | | 87 (4 %) |
| B | 370 | | | 62 (17 %) | | 23 (6 %) | | 51 (14 %) |
| **C** | **2,091** | | | **446 (21 %)** | | **179 (9 %)** | | **358 (17 %)** |
| D | 927 | | | 191 (21 %) | | 72 (8 %) | | 13 (2 %) |
| E | 1,530 | | | 349 (23 %) | | 128 (8 %) | | 105 (7 %) |
| **F** | **2,770** | | | **264 (10 %)** | | **62 (2 %)** | | **303 (11 %)** |
| **G** | **1,227** | | | **254 (21 %)** | | **83 (7 %)** | | **196 (16 %)** |
| **H** | **1,141** | | | **217 (19 %)** | | **80 (7 %)** | | **188 (16 %)** |
| I | 418 | | | 99 (24 %) | | 32 (8 %) | | 188 (45 %) |
| **J** | **2,247** | | | **379 (17 %)** | | **151 (7 %)** | | **372 (17 %)** |
| K | 260 | | | 80 (31 %) | | 35 (1 %) | | 44 (17 %) |
| **L** | **1,032** | | | **184 (18 %)** | | **74 (7 %)** | | **237 (23 %)** |
| Total | 16,311 | | | 2,949 (18 %) | | 1,081 (7 %) | | 2,142 (13 %) |

Bold fonts indicate the clades include more than 1000 genes and 10 % DEGs.

**Reference**

1. Reimand J, Kull M, Peterson H, Hansen J, Vilo J: **g:Profiler--a web-based toolset for functional profiling of gene lists from large-scale experiments**. *Nucleic acids research* 2007, **35**(Web Server issue):W193-200.

2. Chen CK, Ng CS, Wu SM, Chen JJ, Cheng PL, Wu P, Lu MJ, Chen DR, Chuong CM, Cheng HC *et al*: **Regulatory Differences in Natal Down Development between Altricial Zebra Finch and Precocial Chicken**. *Molecular biology and evolution* 2016.
