# Supplementary material for: Identification and evolutionary analysis of long non-coding RNAs in zebra finch
Source: BMC Genomics. 2017 Jan 31;18:117. doi: 10.1186/s12864-017-3506-z (PMC5282891; doi:10.1186/s12864-017-3506-z)
Supplement: Additional file 13: Table S8. — Primer pair sequences used in this study. (DOCX 15 kb) [file 12864_2017_3506_MOESM13_ESM.docx]

Table S8. Primer pair sequences used in Quantitative PCR.

| **Zebra finch** | | |
| --- | --- | --- |
| Transcripts name Forward (F) or Reverse (R) Sequence (5’-3’) | | |
| *CUFF.6222.3* | F | GGCCACCTCCCAAAACCCTG |
|  | R | AACGTGGAAATCCGGCGTCT |
| *CUFF.14902.2* | F | AGCTACAGCATGAAAATCCATCTCC |
|  | R | GGGGGACATTTATCTGGGCCATT |
| *CUFF.19772.1* | F | TGGAGACAAACCAGACGCCA |
|  | R | TCTCACAGTGCTACCTTGGCA |
| **Chicken** | | |
| *CUFF.6222.3* | F | GCATCATTCTTCGTGTTTCCTTCCC |
|  | R | TTTGGGAGGTGGCCAGGGTA |
| *CUFF.14902.2* | F | GCTACAGCATGAAAATCCATCTCC |
|  | R | GGGGGACATTTATCTGGGCCATT |
| *CUFF.19772.1* | F | TGCCCCTGAGAGAGCCAGTT |
|  | R | CGCTGTGCATCTTGCTTTGGG |
